# Tinnitus as a comorbidity to depression and transcranial magnetic stimulation as a treatment for both - case report

**DOI:** 10.1192/j.eurpsy.2022.1914

**Published:** 2022-09-01

**Authors:** P. Marinovic, M. Zivkovic, T. Bagaric, M. Skocic Hanzek, A. Mihaljevic-Peles

**Affiliations:** University Hospital Centre Zagreb, Psychiatry, Zagreb, Croatia

**Keywords:** Antidepressants, Depression, tinnitus, TMS

## Abstract

**Introduction:**

Depressive symptoms are common in individuals with tinnitus, however, the mechanisms of their interaction are not fully understood. There is neurobiological evidence that might help understanding the interplay between tinnitus and depression which, in turn, helps in making the right choice for treating both conditions.

**Objectives:**

This case report describes a 70-year old female patient that presented with tinnitus and depressive symptoms lasting for the past 5 years.

**Methods:**

The patient showed limited treatment results with different antidepressants. The otorhinolaryngologist ruled out any possible somatic causes of her tinnitus. Tinnitus was causing her sleep disturbances, which worsened her everyday functioning that was already quite poor even further.

**Results:**

After being administered with 30 rounds of TMS, her symptoms either completely resolved or at least reached a level that was adequate for her to start functioning normally on a day-to-day basis.

**Conclusions:**

TMS is a technique that provides non-invasive cortical stimulation, more specifically, when used for depression treatment it stimulates the left dorsolateral prefrontal cortex, a brain region synaptically connected to the limbic system involved in mood regulation that is proven to be hypoactive in depression. The limbic system is where tinnitus-related brain networks and regions involved in the pathophysiology of depression overlap. Further research is needed to deepen the understanding of this topic.

**Disclosure:**

No significant relationships.

